# An evaluation of transport mode shift policies on transport-related physical activity through simulations based on random forests

**DOI:** 10.1186/s12966-017-0600-1

**Published:** 2017-10-23

**Authors:** Ruben Brondeel, Yan Kestens, Basile Chaix

**Affiliations:** 1 Inserm, UMR-S 1136, Pierre Louis Institute of Epidemiology and Public Health, Nemesis team, Médecine Saint-Antoine, 27 rue Chaligny, UMR-S 1136, 75012 Paris, France; 2 Sorbonne Universités, UPMC Univ Paris 06, UMR-S 1136, Pierre Louis Institute of Epidemiology and Public Health, Nemesis team, Paris, France; 3 EHESP School of Public Health, Rennes, France; 4 Department of Social and Preventive Medicine, University of Montreal, Montreal, Quebec Canada

**Keywords:** Active transport, Transport, Simulation study, Machine learning, Data integration, Health inequalities, RECORD cohort study, France

## Abstract

**Background:**

Physical inactivity is widely recognized as one of the leading causes of mortality, and transport accounts for a large part of people’s daily physical activity. This study develops a simulation approach to evaluate the impact of the Ile-de-France Urban Mobility Plan (2010–2020) on physical activity, under the hypothesis that the intended transport mode shifts are realized.

**Methods:**

Based on the Global Transport Survey (2010, *n* = 21,332) and on the RECORD GPS Study (2012–2013, *n* = 229) from the French capital region of Paris (Ile-de-France), a simulation method was designed and tested. The simulation method used accelerometer data and random forest models to predict the impact of the transport mode shifts anticipated in the Mobility Plan on transport-related moderate-to-vigorous physical activity (T-MVPA). The transport mode shifts include less private motorized trips in favor of more public transport, walking, and biking trips.

**Results:**

The simulation model indicated a mean predicted increase of 2 min per day of T-MVPA, in case the intended transport mode shifts in the Ile-de-France Urban Mobility Plan were realized. The positive effect of the transport mode shifts on T-MVPA would, however, be larger for people with a higher level of education. This heterogeneity in the positive effect would further increase the existing inequality in transport-related physical activity by educational level.

**Conclusions:**

The method presented in this paper showed a significant increase in transport-related physical activity in case the intended mode shifts in the Ile-de-France Urban Mobility Plan were realized. This simulation method could be applied on other important health outcomes, such as exposure to noise or air pollution, making it a useful tool to anticipate the health impact of transport interventions or policies.

**Electronic supplementary material:**

The online version of this article (doi: 10.1186/s12966-017-0600-1) contains supplementary material, which is available to authorized users.

## Background

Overall physical activity levels are low worldwide, with an estimated 31% of adults physically inactive [1]. Physical inactivity is widely recognized as one of the leading causes of mortality and morbidity due to its impact on several noncommunicable diseases [2, 3]. Therefore, the World Health Organization (WHO) and many governments have adopted health plans to promote regular physical activity [2, 3].

Transport activity can be an important source of regular, incidental physical activity [4–6], making transport policy a promising mean for the promotion of physical activity [7, 8]. Previous studies have evaluated real-world transport policies and interventions in terms of physical activity [8–11]. In two review studies, Scheepers et al. [8] and Petrunoff et al. [11] found mostly positive effects on mode shifts from car to active transport of a range of transport interventions; where Arnott et al. [9] cautioned that there was no evidence for the effectiveness of behavioral interventions, in contrast with structural interventions.

Most previous intervention studies used the transport modes as indicators of transport-related physical activity. Other studies have used more direct indicators such as moderate-to-vigorous physical activity, however, usually based on survey measurements. Intervention studies that evaluate objective measures of physical activity are rare and remain difficult to conduct. Transport interventions on a community scale are often costly, and the evaluation of such interventions (e.g., with before and after assessments) are challenging to design and are themselves costly when relying on assessment methodologies such as accelerometers [12, 13]. Moreover, real-world interventions are often implemented over restricted territories or populations (e.g., one school or company), and their impact may be difficult to generalize to a larger population. Also, intervention studies are restricted to the particular intervention design, making it impossible to determine what would have been the impact of the intervention if it had been implemented in a different way or with a different intensity.

To address these concerns, as a complement of evaluations of real-world interventions, the aim of the present study was to develop and to apply a method to evaluate the impact of transport mode shifts on transport-related physical activity. We propose a simulation approach based on random forest prediction models and apply it to the 2010–2020 transport policy goals of the Ile-de-France Transport Authority (Syndicat des transports d’Île-de-France) and Ile-de-France Regional Council, as described in the 2010 Urban Mobility Plan [14]. The Ile-de-France Transport Authority is responsible of the transport in the French capital region, and includes political representatives of the region and the 9 departments within the region. The Urban Mobility Plan aimed to decrease car and motorbike trips (− 2%) and increase walking (+10%), biking (+10%) and public transport trips (+20%) by a mix of mostly structural interventions (e.g. extra tramway lines, expanding existing metro lines).

To illustrate further the pertinence of the method for transport and public health policies, we evaluated the impact of this transport mode shift policy on the magnitude of inequalities in transport-related physical activity by educational level. Previous research showed that people with a low educational level have lower physical activity levels [15]; and transport interventions, like other health-related interventions, have the potential to enlarge existing social inequalities [10, 16]. It is therefore important for policy makers to be able to anticipate this type of unwanted side-effects.

## Methods

### Study population

The Global Transport Survey (‘Enquête Globale Transport’, EGT) is a household travel survey conducted every 10 years in Île-de-France, the French capital region. The main purpose of the survey is to inform local authorities and transport planners on transport behavior in Île-de-France. The last EGT survey, approved by the French Data Protection Authority, was conducted in 2010 by the Ile-de-France Transport Authority (STIF) and the Regional and Interdepartmental Direction for Equipment and Planning (DRIEA). During face-to-face interviews with members of randomly selected households, data were collected for all the trips made during the day before the interview. For this study, we selected participants between 35 and 83 years old to match the age range of the RECORD data that were used to predict the transport-related physical activity (see section Measures and Definitions). This resulted in a dataset of 82,084 trips made by 21,332 people.

### Measures and definitions

There were no accelerometer data in the EGT sample. Therefore, the measure of transport-related moderate to vigorous physical activity (T-MVPA) was introduced in the dataset by the integration of the EGT and the RECORD GPS Study datasets. The RECORD dataset was used to develop a prediction model for T-MVPA which was then applied to the EGT dataset.

The RECORD GPS Study [4, 13, 17, 18], as a subsample of the RECORD Cohort Study [19–24], collected transport behavior and accelerometer data for 236 participants during 7 days, resulting in the observation of 7138 trips. All participants resided in Ile-de-France and were between 35 and 83 years old. A full description of the study design can be found in Additional file 1. In the RECORD GPS Study, trips and transportation modes were detected by a prompted recall survey, i.e. a survey enhanced with the results of an algorithm detecting trips and activity places [25] based on the data of a hip worn GPS. A minute of T-MVPA was defined as a minute during which the 3-axis vector magnitude was higher than 2690 based on the tri-axial GT3X+ accelerometer data (the definition of moderate-to-vigorous physical activity by Sasaki and colleagues [26]) during transport. Accelerometers worn at the hip underestimate physical activity during biking trips. Therefore, we used an estimate of biking physical activity from the compendium of Ainsworth [27], i.e., all minutes during biking trips were considered as minutes of T-MVPA.

The data integration consisted of predicting T-MVPA in EGT based on the data of the RECORD GPS Study [28], using a random forest prediction model [29]. Random forest is machine learning prediction model based on decision trees that can be used for predicting both continuous and categorical variables [30]. The random forest model included 45 variables to predict the accelerometer based T-MVPA, including trip characteristics (e.g., transportation mode, duration), personal characteristics (e.g., age and educational level), personal transport accessibility characteristics (e.g., possession of a motorized vehicle) and area transport accessibility characteristics for the residence and the departure and arrival location of each trip (e.g., distance to nearest transport station). The variables were selected because they were hypothesized to be predictive of T-MVPA and available in both datasets. Additional file 2 presents the full list of variables used for the data integration. The R^2^ for this model was 0.67, indicating that the model could very accurately predict the accelerometer based T-MVPA during a trip observed in the RECORD study. There was no specific validation method (e.g. cross-validation) included, since the random forest method has a built in validation method [30]. With the EGT data originating from the same population and the same region, we hypothesized that the model provided accurate predictions of T-MVPA for the trips in the EGT dataset. The data integration process was previously described in detail [28].

Three categories of educational level were considered: ‘no diploma of secondary education’, ‘diploma of secondary education or lower tertiary education’, and ‘diploma of higher tertiary education’. The transportation mode variable consisted of four categories: ‘walking’, ‘bicycle’, ‘private motorized’, and ‘public transport’. Trips with non-walking modes that also included ‘walking’ were categorized on the basis of the non-walking mode.

### Statistical analysis

Three scenarios of transportation mode shifts were considered. The main scenario reflected the goals of the local transport authority stated in the 2010 Ile-de-France Urban Mobility Plan. The goals were to increase public transport use by 20% and walking and biking by 10% between 2010 and 2020 [14]. These goals were formulated by the local transport authority under the assumption of a 7% increase in the number of trips in Ile-de-France (due to an increase in population size and a changing demographic structure) [14]. In the present study, the aim was to investigate the impact of modal shifts on the physical activity of the current population, therefore, independent of the overall increase in the number of trips. As detailed in Additional file 3, the goals in modal shifts - after disregarding the overall increase in the number of trips - corresponded to an increase by 11.8% of public transport trips and by 2.5% of walking and biking trips. For our sample including a total of 82,084 trips, these modal shifts resulted in 1386 more public transport trips, 688 more walking trips, 30 more biking trips, and consequently 2104 less private motorized trips (i.e. car and motorbike trips). To investigate the potential impact of more ambitious policies, two extra scenarios were tested doubling and tripling these modal shifts.

In this main simulation however (i) all these changes (e.g., related to public transport, walking, and biking) were implemented simultaneously; and (ii) the flow of mode shifts from private motorized modes to more active modes was pre-determined. We thus ran a complementary simulation where (i) we assessed separately the impact of the three components: the change in the number of public transport trip, the number of walking trips and the number of biking trips and (ii) where we did not specify the origin mode from which changes were made (e.g., both car trip and walking/biking trips could be changed to public transport trips, the latter reflecting an unwanted effect of the policy).

The simulation process for the scenarios consisted of three consecutive steps. In a first step, 2104 private motorized transport trips were selected and changed into one of the three other modes: public transport, walking, or biking. To simulate the intended mode shifts described in the mobility plan (11.8% increase in public transport trips and 2.5% increase in both walking and biking trips), 3.33% (*n* = 1386) of the private motorized trips in the EGT data set had to be selected and changed into public transport, 1.65% (*n* = 688) into walking trips and 0.07% (*n* = 30) into biking trips. The trips were selected in function of the likelihood that the trips could be performed by these modes. The likelihood was calculated by a random forest model based on the original data. This prediction model took into account variables predictive of the transport mode, e.g. distance between departure and arrival and age of respondent (see Table 1 (column 1) for the full list of variables). The random forests prediction method [29] is based on the decision tree method. Decision trees classify observations in subsequent steps (nodes), aiming to obtain homogenous groups in terms of the outcome variable, in this case, the transportation mode. To avoid overfitting, a random forest only uses a subsample of all available variables at each node, and it uses a large number of trees, each tree grown on a subsample of the observations (about 68%). After growing a tree, a prediction is made for those observations that were not used in that particular tree. At the end of the algorithm, there are predictions for each observation from about 32% of the trees. For example, a particular trip observed as a public transport trip, could have been predicted as a public transport trip in 60% of the trees, a private motorized trip in 20% of the trees, a biking trip in 15% of the trees and a walking trip by 5% of the trees. These proportions were used in the simulation model as variables indicating the likelihood of the observed private motorized trips to be done by the three other transportation modes. The likelihood variables were then rescaled so that the mean likelihood corresponded to the intended modal shift. For example, the mean likelihood of the private motorized trips to be done by walking was 0.11 (i.e. the mean proportion of trees predicting observed private motorized trips as walking trips) according to the random forest model, whereas the proportion of the private motorized trips needed to shift in order to obtain the 2.5% increase in walking trips intended in the Urban Mobility Plan was only 3.33%. The likelihood variable was therefore rescaled so that its mean would be equal to 0.033 instead of 0.11. This rescaling relied on a transformation of the probabilities to the logit scale, a shift in the logit scale, and then a transformation back to the probability scale, in order to avoid probabilities out of the [0; 1] range. Trips were then selected by taking random binomial samples (1 = selected and 0 = not selected), using the values of the likelihood variable as the probability for each trip of being selected for a mode shift.

**Table 1:** Variables used to predict transportation mode (TM), duration of trip (D), and transport-related MVPA (MVPA)

	TM	D	T- MVPA
Minutes of T-MVPA per trip^a^			X
Duration of the trip (in minutes)^b^		X	X
Speed based on duration and straight-line distance^b^			X
Transportation mode^b^	X	X	X
Trip characteristics			
Time of the day at departure^b^	X	X	X
Day of the week at departure^b^	X	X	X
Rush hour or not at departure: from 8 am to 11 am and from 4 pm to 7 pm^b^	X	X	X
Straight-line distance from departure point to arrival point^c^	X	X	X
Personal variables			
Age^c^	X	X	X
Gender^c^	X	X	X
Work situation (employed, unemployed, retired, other)^c^	X	X	X
Personal educational level^c^	X	X	X
Household income^c^	X	X	X
Spatial access to public transport from the trip departure, trip arrival, and residence (3 separate sets of variables)			
Street network distance to nearest public transport station from residence^d^	X	X	X
Street network distance to nearest train station^d^	X	X	X
Street network distance to nearest metro station^d^	X	X	X
Street network distance to nearest tram station^d^	X	X	X
Street network distance to nearest bus station^d^	X	X	X
Other contextual characteristics at the trip departure, trip arrival, and residence (3 separate sets of variables)			
Educational level in the area^e^	X	X	X
Number of destinations in the area^e^	X	X	X
Number of intersections in the area^e^	X	X	X
Surface of parks in the area^e^	X	X	X
Population density in the area^e^	X	X	X
Place located in Paris, in the other counties adjacent to Paris, or in the other counties non-adjacent to Paris	X	X	X

^a^Accelerometer information in RECORD or predicted time in EGT

^b^Information from the mobility survey in RECORD and in EGT

^c^RECORD and EGT questionnaires

^d^Shortest street network distance determined with ArcGIS from the residence or from the departure/arrival of each trip geocoded at the address level in RECORD or at the center of a 100 m square in EGT

^e^The area around the residence or departure or arrival point of each trip was defined with ArcGIS as a 1 km buffer following the street network, and information was aggregated at the level of this buffer; MVPA: moderate to vigorous physical activity

After selecting 2104 trips and changing the transport modes, the duration of the trip was predicted for the trips to which a new transportation mode was attributed in step 1. The prediction was based on a random forest model for the duration of trips in the original EGT data (i.e. before the simulated modal shift). In a final step, the T-MVPA was predicted for the trips with a changed transportation mode and duration using the same prediction model as for the data integration, i.e., a random forest model for T-MVPA based on the RECORD GPS data. The simulation of each scenario was repeated 100 times to avoid the impact of random sampling error in the results. Table 1 presents all the variables used in the three random forest models for the transportation mode, duration, and T-MVPA. The scripts for all the analyses with R (v3.3.0) [31] can be found in Additional file 4.

## Results

Table 2 presents the predicted daily T-MVPA for the observed transport behavior by educational level and by transportation mode. The mean T-MVPA for the complete sample was 18.98 min per day (95% CI = 18.94; 19.02). Most of the observed T-MVPA was related to walking trips (6.81 min, CI = 6.79; 6.83) and public transport trips (6.79 min, CI = 6.77; 6.80), followed by private motorized transport trips (4.21 min, CI = 4.20; 4.21) and biking trips (1.17 min, CI = 1.16; 1.18). The mean T-MVPA was 17.52 (95% CI = 17.48; 17.55), 18.63 (95% CI = 18.59: 18.67) and 21.05 (95% CI = 21.01; 21.08) minutes per day for people with a low, medium, and high educational level respectively. These inequalities originated mainly from the inequalities in T-MVPA related to public transport and biking trips.

**Table 2:** Predicted T-MVPA over 1 day for observed transport behavior by educational level and transport mode

	Total	E1	E2	E3
All modes	19.0	17.5	18.6	21.1
Walking trips	6.8	6.9	6.2	7.1
Biking trips	1.2	0.8	1.1	1.7
Public Transport trips	6.8	5.7	6.8	8.1
Private motorized transport	4.2	4.1	4.5	4.1

Total number of persons = 21,332; Total number of trips = 82,084, *T-MVPA* transport-related moderate-to-vigorous physical activity expressed in minutes per day; *Total* mean T-MVPA for complete population, *E1* no diploma of secondary education, *E2* Diploma of secondary education or 2 years or less of University education; *E3* Diploma of 3 years or more of University education

Table 3 presents the gain in T-MVPA due to the simulated transport mode shifts from the 2010 Ile-de-France Urban Mobility Plan, by transportation mode and educational level. The first model, reflecting the goals of the local transport authority, resulted in a mean gain of 1.9 min of T-MVPA. Doubling and tripling the transport mode shifts resulted in gains in T-MVPA of 2.9 min and 4.0 min respectively. Most of the gain originated from public transport trips.

**Table 3:** Gains in T-MVPA per day after simulated transport mode shifts by educational level and transport mode

	Model 1				Model 2				Model 3			
	Total	E1	E2	E3	Total	E1	E2	E3	Total	E1	E2	E3
All modes	1.9	1.6	1.9	2.2	2.9	2.6	3.0	3.3	4.0	3.6	4.1	4.5
Walking trips	0.8	0.8	0.8	0.9	1.1	1.1	1.1	1.2	1.5	1.4	1.4	1.5
Biking trips	0.1	0.0	0.0	0.1	0.1	0.1	0.1	0.2	0.2	0.1	0.1	0.2
Public transport trips	1.2	1.0	1.2	1.4	2.1	1.8	2.2	2.5	3.1	2.7	3.2	3.5
Private motorized trips	−0.2	−0.1	−0.1	−0.2	−0.4	−0.4	−0.4	−0.5	−0.7	−0.6	−0.7	−0.8

Total number of persons = 21,332; Total number of trips = 82,084, *T-MVPA* transport-related moderate-to-vigorous physical activity expressed in min per day, *Total* gain in mean T-MVPA for complete population, *E1* no diploma of secondary education, *E2* Diploma of secondary education or 2 years or less of University education, *E3* Diploma of 3 years or more of University education

The gain in T-MVPA is the largest for people with the highest educational level, and the lowest for the people with the lowest educational level. Especially, the T-MVPA gained from public transport trips further enlarged the existing inequalities in T-MVPA by educational level.

Table 4 presents the daily T-MVPA by transportation mode, before and after mode shifts in four complementary simulations. In these complementary analyses, each mode shift in the Ile-de-France Urban Mobility Plan was simulated separately. For example, public transport trips were augmented by 11.8% in the ‘Public transport’ simulation. In contrast to the main simulation, not only private motorized trips, but also walking and biking trips could be changed into public transport trips depending on the trips’ likelihood of being performed by public transport. For each simulation, Table 4 shows the intended positive effects and the unintended reverse effects, i.e., the loss of physical activity for the alternative transportation modes. For example, in the public transport simulation, the daily T-MVPA related to public transport increased by 0.86 min; the overall gain, however, was 0.52 min due to the decrease in T-MVPA related to walking, biking and private motorized trips.

**Table 4:** Gains in T-MVPA per day after simulated transport mode shifts by educational level and transport mode – modeling transport shifts separately

	Observed	Transportation mode shift simulations^a^			
		Public transport	Walking	Biking	Priv. motorized
Total T-MVPA	19.0	+ 0.52	+ 0.16	+ 0.02	+ 0.82
T-MVPA in walking trips	6.8	- 0.13	+ 0.29	0.00	+ 0.42
T-MVPA in biking trips	1.2	- 0.08	- 0.02	+ 0.04	+ 0.01
T-MVPA in public transport trips	6.8	+ 0.86	- 0.05	- 0.01	+ 0.63
T-MVPA in private motorized trips	4.2	- 0.14	- 0.06	0.00	- 0.25

T-MVPA: transport-related moderate-to-vigorous physical activity expressed in min per day; Priv. motorized: private motorized transport

^a^Complementary simulations, with one simulation related to each transportation mode, e.g. simulation public transport involved 11.8% more public transport trips. The original transport mode could be any of the three other modes (compared to strictly ‘private motorized’ Model 1, Model 2 and Model 3)

## Discussion

### Main results

This study underlines the importance of transport for reaching daily physical activity levels. People between 35 and 83 years old residing in the Ile-de-France region had an average of 19 min of daily T-MVPA, largely obtained during walking trips and public transport trips. Transport interventions to promote physical activity traditionally focus on walking and biking [11]. Recently, public transport has been found to also contribute significantly to population physical activity levels [4, 11, 28, 32]. The results in this study confirm this finding, with a mean of 6.8 min of daily MVPA related to public transport, corresponding to 36% of the total daily T-MVPA.

Applying on our sample the intended transport mode shift described in their 2010 Urban Mobility Plan from the Transport Authority of Ile-de-France and Ile-de-France Regional Council, resulted in a predicted 1.9 min per day increase of T-MVPA. Doubling (model 2) and tripling (model 3) the intended transportation shifts resulted in increases of 2.9 min per day and 4 min per day respectively. This illustrates that more ambitious plans might be necessary to obtain significant increases in T-MVPA at a population level. It is however unclear how realistic these more ambitious plans are in the short term from an infrastructural perspective. For example, the increased use of the public transport system in models 2 (+24%) and 3 (+35%) might be unrealistic. Strategies with more focus on walking and biking might be needed to obtain these higher levels of transport-related physical activity.

The complementary simulations (see Table 4) simulated the mode shifts separately; representing the different approaches in the Urban Mobility Plan: promoting public transport, walking and biking, and discouraging private motorized transport. The main finding of these analyses was the reverse effects that were not observable in the main simulation. For example, increasing the number of public transport trips not only lead to less private motorized trips, but also decreased the number of trips performed by walking or biking; the latter changes reduced the physical activity during these trips. These results suggest that public transport should explicitly be promoted as an alternative for private motorized transport exclusively, to limit these reverse effects.

Disparities in T-MVPA by educational level were observed before the simulations; and such disparities were larger after the simulated transportation modes shifts. Public transport changes were the main contributor to the increase in educational inequalities. One explanation is that public transport trips are more likely to occur in or nearby the city center of Paris, where also relatively higher educated people live. In our sample, 25% of the highest educated people live in Paris, compared to only 6% of the lowest educated.

The increases in the educational disparities were probably even underestimated in the simulations. The differential impact of the mode shifts by educational level was completely due to the characteristics of the trips (e.g., length of the trip) made by each group. In real-life interventions, not only trip characteristics but also uptake of, access to, and compliance with the intervention are likely to be different between educational groups [16]. Some of these factors were accounted for in our model defining the probability of change (e.g., work situation, geographic location of the residence, spatial access to services and public transport). However, since it is unlikely we included all factors contributing to a weaker impact of an intervention or policy among the lower educated, we can expect that educational inequalities after an intervention would likely be larger than predicted in this study. Too often, social disparities are neglected in the design and evaluation of active transport interventions [33]. The simulation method presented in this paper may help policy makers avoid these unwanted side-effects during the design phase of transport policies and interventions.

### Strengths and limitations

The work presented in this article can be related to health impact studies, in particular studies on the impact of active transport on mortality and morbidity outcomes (e.g. type II diabetes) by intermediary variables such as physical activity, traffic injuries, traffic related noise and air pollution [34]. Health impact models in the literature include HEAT (Health Economic Assessments Tools) [35], ITHIM (Integrated Transport and Health Impact Modelling tool) [36], or DYNAMO-HIA [37, 38]. This study focused on one part of the causal chain in a health impact assessment, namely the impact of the transport mode choice on physical activity. Health impact models are therefore richer in the number of health outcomes, and offer a more holistic view of the health impact of mobility plans. However, the model presented in this article is more complete on the specific link between transport mode and physical activity in two ways, and the integration of this model into health impact models may therefore significantly increase their accuracy and efficiency. First, using of a high variety of predictors in combination with the random forest prediction model enhanced significantly the accuracy of the prediction model. Secondly, the pre-simulation probabilities of each trip to be performed by an alternative mode (e.g. the probability of a private motorized trip to be done by walking) allowed for more likely transport mode shifts. The third, and perhaps most important improvement in this simulation model, is the introduction of accelerometer data. To our knowledge, all previous health impact studies used survey data and were therefore limited to a strict separation between active transport (i.e. walking and cycling) and non-active transport (public and private motorized transport). Using accelerometer data allows for transport-related physical activity during so-called non-active trips, in particular public transport trips.

Simulation studies cannot replace intervention studies and are to be considered as indicative rather than empiric [39]. There are many unknown variables in a real-life setting that cannot be simulated such as the adaptation of participants to an intervention, the longer term effects, unintended changes in other variables that are important for the outcome, etc. However, we argue that well-designed simulations in combination with rapidly enhancing machine learning algorithms and the growing number of data sources, can be complementary to intervention studies. Simulations have the great advantage of being very cost-efficient, while allowing for the comparison of a multitude of intervention scenarios.

The most important limitation of this simulation is that we had to assume that transport scenarios could only affect the transportation mode used in a trip, but that it could not influence the choice to make a trip or the destination of the trip itself. For example, a successful transport intervention may motivate people to choose a destination further away than the current destination if this further destination has more to offer.

Only transport-related physical activity has been analyzed in this study. However, an increase in transport-related physical activity might lead to a decrease of physical activity in other domains (e.g. leisure), resulting in no increase or a limited increase in the total physical activity. Previous studies have found no evidence of this ‘compensation’ theory [6]; but it cannot be excluded that compensation does play a role in certain subpopulations such as older adults. Also, the studies reporting on the compensation of increased transport-related physical activity in other domains were all based on survey data. More studies on objective physical activity data are needed.

The collection of accelerometer measures of transport-related physical activity is very expensive for large samples. Survey measures of physical activity are prone to memory biases and they only approximate physical activity by indirect indicators (e.g. ‘minutes of walking during one week’). We therefore used data integration to add an accelerometer measure of transport-related physical activity to the large survey dataset [16, 28]. Even though the prediction model had a high accuracy, real measured accelerometer data would definitely be preferable. The advantage of using this outcome over survey data is the detail it provides. For example, public transport trips are clearly not fully inactive periods of time, since the person has to walk to and from the public transport station. The predicted measure of MVPA used in this study can capture this type of physical activity, making it an interesting measure for large-scale studies for which real accelerometer data is unavailable.

## Conclusions

The method presented in this study calculated the impact of the intended transport mode shifts in the regional Urban Mobility Plan (Paris region, France) on T-MVPA, an important indicator of physical activity. The results showed a mean increase of 2 min per day of T-MVPA due to the mode shifts from private motorized modes (i.e. car and motorbike) to more active modes (i.e. walking, biking and public transport). However, the results also indicated an increase in the inequalities by educational level already present in the study population. The method could be extended to other settings. We also aim to apply it to other important transport health-relevant outcomes such as exposure to noise or air pollution (using such environmental data collected with wearable sensors in a population sample), making it a useful tool to estimate the health impact of transport interventions or policies during the design phase.

## Additional files

Additional file 1: Description of the RECORD GPS Study. (PDF 72 kb)

Additional file 2: Overview of the variables used in the data integration. (PDF 44 kb)

Additional file 3: Implications of the 2010 Ile-de-France Urban Mobility Plan objectives for the study sample. (PDF 105 kb)

Additional file 4: R-scripts: simulation models and data integration. (PDF 137 kb)

## Abbreviations

CI: Confidence interval
EGT: Enquête globale transport
GPS: Global positioning system
MVPA: Moderate-to-vigorous physical activity
RECORD: Residential environment and coronary heart disease
T-MVPA: Transport-related moderate-to-vigorous physical activity
WHO: World Health Organization
