# Supplementary material for: An evaluation of transport mode shift policies on transport-related physical activity through simulations based on random forests
Source: Int J Behav Nutr Phys Act. 2017 Oct 23;14:143. doi: 10.1186/s12966-017-0600-1 (PMC5651637; doi:10.1186/s12966-017-0600-1)
Supplement: Supplementary file 1 — Description of the RECORD GPS Study. (PDF 72 kb) [file 12966_2017_600_MOESM1_ESM.pdf]

Description of the RECORD GPS Study

April 26, 2017

The participants in the RECORD Study (Residential Environment and CORonary heart Disease) were recruited during preventive health checkups in 20072008, and were born in 19281978.[1,2] Every participant residing in 112 pre-selected municipalities of the Ile-de-France Paris region at baseline presenting at the IPC Medical Center for a health checkup was invited to enter the RECORD Study.[3,4] The selected municipalities of the Ile-de-France region included a broad range of municipalities in terms of household income and urbanicity degree.

In the second wave of the study (2011-2012),[5–8] 410 RECORD participants were invited to enter the RECORD GPS Study.[2,9] Of these, 236 accepted to participate. Participants wore a BT-Q1000XT GPS (QStarz) and a GT3X+ accelerometer (The Actigraph) on the right hip with a dedicated elastic belt, for the recruitment day and 7 additional days, all day long from wake up to bedtime. The participants had to fill out a travel diary by reporting their activity places over the 7-8 days, each time with arrival and departure times.

The GPS data were collected every 5 seconds. After linear interpolation of the missing data, the GPS data were analyzed with an algorithm (ArcGIS Python script) that identified all of the activity locations of the participants (any activity at a stationary location) from the accumulation of GPS points over 7 days.[10] Based on these outputs of the algorithm, the Mobility Web Mapping application was then used to visualize the activity patterns on a map per participant per day. The Mobility Web Mapping application was designed by the University of Montreal. The application was used to survey the participants on the activity performed at each visited location and on the modes used in each trip. The survey operator could report activity locations and trips undetected by the algorithm and could modify/remove detected visits to locations that were inaccurate or incorrect. This procedure resulted in the identification of 7138 trips for 229 participants. Written informed consent was obtained from all participants. The RECORD GPS Study was approved by the French Data Protection Authority.

References

[1] Brondeel R, Weill A, Thomas F, Chaix B. Use of healthcare services in the residence and workplace neighbourhood: the effect of spatial accessibility

to healthcare services. Health Place. 2014;30:127–133.

[2] Chaix B, Kestens Y, Duncan S, Merrien C, Thierry B, Pannier B, et al. Active transportation and public transportation use to achieve physical activity recommendations? A combined GPS, accelerometer, and mobility survey study. Int J Behav Nutr Phys Act. 2014;11(1):124.

[3] Chaix B, Bean K, Daniel M, Zenk SN, Kestens Y, Charreire H, et al. Associations of Supermarket Characteristics with Weight Status and Body Fat: A Multilevel Analysis of Individuals within Supermarkets (RECORD Study). PLoS ONE. 2012;7(4):e32908.

[4] Van Hulst A, Thomas F, Barnett T, Kestens Y, Gauvin L, Pannier B, et al. A typology of neighborhoods and blood pressure in the RECORD Cohort Study. J Hypertens. 2012;30:1336–1346.

[5] Chaix B, Kestens Y, Bean K, Leal C, Karusisi N, Meghiref K, et al. Cohort profile: Residential and non-residential environments, individual activity spaces and cardiovascular risk factors and diseases-The RECORD cohort study. Int J Epidemiol. 2012;41(5):1283–1292.

[6] Chaix B, Kestens Y, Perchoux C, Karusisi N, Merlo J, Labadi K. An Interactive Mapping Tool to Assess Individual Mobility Patterns in Neighborhood Studies. Am J Prev Med. 2012;43(4):440–450.

[7] Leal C, Bean K, Thomas F, Chaix B. Multicollinearity in Associations Between Multiple Environmental Features and Body Weight and Abdominal Fat: Using Matching Techniques to Assess Whether the Associations are Separable. Am J Epidemiol. 2012;175(11):1152–1162.

[8] Perchoux C, Kestens Y, Thomas F, Van Hulst A, Thierry B, Chaix B. Assessing patterns of spatial behavior in health studies: Their sociodemographic determinants and associations with transportation modes (the RECORD Cohort Study). Soc Sci Med. 2014;p. 64–73.

[9] Chaix B, Kestens Y, Duncan DT, Brondeel R, Méline J, El Aarbaoui T, et al. A GPS-based methodology to analyze environment–health associations at the trip level: case-crossover analyses of built environment effects on walking. Am J Epidemiol. (In press);.

[10] Thierry B, Chaix B, Kestens Y. Detecting activity locations from raw GPS data: a novel kernel-based algorithm. Int J Heal Geogr. 2013;12(14).
