# Supplementary material for: An evaluation of transport mode shift policies on transport-related physical activity through simulations based on random forests
Source: Int J Behav Nutr Phys Act. 2017 Oct 23;14:143. doi: 10.1186/s12966-017-0600-1 (PMC5651637; doi:10.1186/s12966-017-0600-1)
Supplement: Supplementary file 2 — Overview of the variables used in the data integration. (PDF 44 kb) [file 12966_2017_600_MOESM2_ESM.pdf]

Supplementary material 2: Overview of the variables used in the data integration

Integrated (predicted) variable
Minutes of transport-related moderate-to-vigorous physical activity (T-MVPA) per trip [a]
Personal variables
Household income [b]
Personal education level [b]
Age [b]
Gender [b]
Work situation (employed, unemployed, retired, other) [b]
A motorized vehicle available in the household [b]
A car available in the household [b]
A motorbike available in the household [b]
In possession of a public transport pass [b]
Spatial access to public transport at the residence
Street network distance to nearest public transport station from residence [c]
Street network distance to nearest train station [c]
Street network distance to nearest metro station [c]
Street network distance to nearest tram station [c]
Street network distance to nearest bus station [c]
Other residential neighborhood characteristics
Educational level in the residential neighborhood [d]
Number of destinations in the residential neighborhood [d]
Number of intersections in the area [d]
Area size of parks in the area [d]
Population density in the area [d]
Address located in Paris, or in the other counties adjacent to Paris, or in the other counties non-adjacent to Paris
Trip characteristics
Transportation mode [e]
Duration of the trip in minutes [e]
Time of the day at departure [e]
Day of the week at departure [e]
Rush hour or not at departure: from 8am to 11am and from 4pm to 7pm [e]
Straight-line distance from departure address to arrival address [e]
Speed based on duration and straight-line distance [e]
Trip departure and arrival location characteristics (2 separate sets of variables)
Distance to nearest train station [c]
Distance to nearest metro station [c]
Distance to nearest tram station [c]
Distance to nearest bus station [c]
Distance to nearest public transport station [c]
Educational level in the area [d]
Number of intersections in the area [d]
Number of destinations in the area [d]
Area size of parks in the area [d]
Population density in the area [d]
Address located in the city center or not (i.e., in Paris as opposed the other parts of Ile-de-France Region)

[a] Accelerometry information in RECORD or predicted time in EGT; [b] RECORD and EGT questionnaires; [c]Shortest street network distance determined with ArcGIS from the residence or from the departure/arrival of each trip geocoded at the center of a 100 m square; [d] The area around the residence or departure or arrival point of each trip was defined with ArcGIS as a 1 km buffer following the street network, and information was aggregated at the level of this buffer; [e]Information from the mobility survey in RECORD and in EGT.
