# Supplementary material for: An evaluation of transport mode shift policies on transport-related physical activity through simulations based on random forests
Source: Int J Behav Nutr Phys Act. 2017 Oct 23;14:143. doi: 10.1186/s12966-017-0600-1 (PMC5651637; doi:10.1186/s12966-017-0600-1)
Supplement: Supplementary file 3 — Implications of the 2010 Ile-de-France Urban Mobility Plan objectives for the study sample. (PDF 105 kb) [file 12966_2017_600_MOESM3_ESM.pdf]

Implications of the 2010 Ile-de-France Urban Mobility Plan objectives for the study sample

April 26, 2017

In the article ‘An evaluation of transport mode shift policies on transport-related physical activity through simulations based on random forests’ (Brondeel, Kestens, and Chaix), the Ile-de-France Urban Mobility Plan was evaluated on its impact on physical activity due to the aimed transport mode shifts.

The Ile-de-France Urban Mobility Plan included the aim to increase public transport trips by 20%, to increase both walking and biking by 10% and to decrease motorized transport trips by 2% between 2010 and 2020.[1] These numbers took into account an overall increase in trips of 7% during this period of time.

In this study, the goal was to predict the impact of the mobility plan on a specific population, under the hypothesis that the number of trips, the departure locations, and the arrival locations would not change. Therefore, we had to adapt the objectives of the mobility plan to a scenario with no overall increase. We calculated first the relative modal shifts between 2010 and 2020, and then calculated the percentage changes, under the condition that the total number of trips did not change.

Table 1: Calculation of modal shifts in stable population

	Public trans.	Priv. motor [a]	Walking / Biking
Mobility plan			
Aims in relative numbers	+20 %	-2 %	+10 %
Absolute number of trips per week day			
2010	8.30 million	16.10 million	16.60 million
2020	9.96 million	15.78 million	18.26 million
Relative number of trips per week day			
2010	20.24 %	39.27 %	40.49 %
2020	22.64 %	35.86 %	41.50 %
Modal shift	+ 2.39 %	- 4.16 %	+ 1.77 %
Within class percentages			
	+ 11.8 %	- 8.7 %	+ 2.5 %

[a] Priv. motor: private motorized, i.e. car and motorbike

Table 1 presents the different steps in the calculations. Take for example the public transport trips (first column). The increase of 20% in public transport trips is equal to an increase of 1.66 million trips (from 8.3 million to 9.96 million). This represents a shift in the relative share of public transport trips of 2.39% percentage points (from 20.24% to to 22.64%). Applying this shift to the 2010 trips, it implicated an increase in absolute numbers of 0.98 million (from 8.3

million to 9.28 million). The 0.98 million trips equal 11.8% of the 8.3 million observed trips, which is the percentage change we used in the main scenario of this paper.

In Table 2 we presented the implications of these modal shifts for our study sample. Take for example public transport trips. For the 21332 participants in our sample, 11744 trips were made by public transport per day. An increase of 11.8% implicated 1386 extra trips made by public transport, previously made by private motorized transport. In the paper, we also examined more ambitious plans by doubling and tripling the transport mode shifts, to see how much extra effect these scenarios would have on physical activity. These two extra scenarios were presented in the last two rows. Note that the data used in this paper originated from the same data set on which the Ile-de-France Urban Mobility Plan is based. We made, however, a selection of people between 35-83 years old, so that the sample corresponded to the RECORD sample.

Table 2: Modal shift applied on study sample

	Public trans.	Priv. motor[a]	Walking [c]	Biking [c]
Observed number of trips				
Observed in 2010	11744	41600	27530	1210
Aims in relative numbers				
Applied mobility plan	+ 11.8 %	- 5.1 % [b]	+ 2.5 %	+ 2.5 %
Differences per scenario				
Scenario 1 - Mobility plan	+ 1386	- 2104	+ 688	+ 30
Scenario 2 - Mobility plan * 2	+ 2772	- 4208	+ 1376	+ 60
Scenario 3 - Mobility plan * 3	+ 4158	- 6312	+ 2064	+ 90

[a] Priv. motor: private motorized, i.e. car and motorbike; [b] This percentage does not correspond to the -8.7% calculated in Table 1, since the distribution of the transport modes is different for our study population compared to the total population. The combined increase in public transport, walking and biking trips correspond to a 5.1% decrease in car trips; [c] Since the Ile-de-France Urban Mobility Plan reported an aim for both modes together, we needed to assume that the increase would be equal (in relative numbers) for both modes.

Due to a different relative distribution of transport modes in our study population compared to the total population, we could not apply all transport shifts as calculated in Table 1. An increase of 11.8% in public transport trips and increases of 2.5% in both walking and biking trips correspond to an decrease in motorized transport trips of 5.1% instead of 8.7% in the total population. Since this paper had an interest in physical activity, we decided to use the exact percentage points for the active transport modes (walking, biking and public transport), and adjusted the shift the motorized trips accordingly.

References

[1] Syndicat des transports d'Ile-de-France. Plan de dplacements urbains, Ile-de-France; 2014. Available from: http://pduif.fr/IMG/pdf/pduif_2014.pdf.
