# Supplementary material for: An evaluation of transport mode shift policies on transport-related physical activity through simulations based on random forests
Source: Int J Behav Nutr Phys Act. 2017 Oct 23;14:143. doi: 10.1186/s12966-017-0600-1 (PMC5651637; doi:10.1186/s12966-017-0600-1)
Supplement: Supplementary file 4 — R-scripts: simulation models and data integration. (PDF 137 kb) [file 12966_2017_600_MOESM4_ESM.pdf]

```
##########################################################################
# Supplementary material S4 for the article:
# Brondeel R, Kestens Y, Chaix B. An evaluation of transport mode shift policies on
transport-related physical activity through simulations based on random forests'.
# The results of these data analyses illustrated the potential impact of
# successful transport interventions on transport-related MVPA (T-MVPA)
#
# These scripts are not the complete scripts used for this article,
# but only illustrative of all the different steps in the
# data integration and simulation process.
# Questions can be directed to the first author (Ruben.Brondeel@gmail.com)
##########################################################################

############################
# R version 3.3.3 (2017-03-06) -- "Another Canoe"
# Copyright (C) 2017 The R Foundation for Statistical Computing
# References can be found below, or obtained by running
# citation('randomForest'), citation('data.table') and
# citation('mice') in your R-console

library(randomForest)
library(data.table)
library(mice)

############################
# Read in EGT data and convert to data tables (instead of data frames)

path <- '…./your_directory/'
rec <- data.table(read.csv(paste0(path, "1. RECORD.csv")))
egt <- data.table(read.csv(paste0(path, "1. EGT.csv")))

############################
# A. Random Forest prediction models
#    These models will later be used for the data integration
#    and the simulations.
#    (for more details on the data integration see
#    Brondeel R, Pannier B, Chaix B. Associations of socioeconomic
#    status with transport-related physical activity: combining a
#    household travel survey and accelerometer data using random forests.
#    J Transp Health. In press. http://dx.doi.org/10.1016/j.jth.2016.06.002)

# 1. Transportation mode prediction model in EGT dataset
#      This model is later used to calculate the probability of the
#      transportation modes for each trip

form.tm <- formula(mode_trans1 ~
                    time_of_day + day_trip + rush_hour + dist_ld +
                    age + homme + rvnu + emploi_sim + nivetude_sim +
                    dist_train_dep + dist_metro_dep +
                    dist_tram_dep + dist_bus_dep +
```

```
                                   dist_train_arr + dist_metro_arr +
                                   dist_tram_arr + dist_bus_arr +
                                   dist_train_res + dist_metro_res +
                                   dist_tram_res + dist_bus_res +
                                   dist_pt_res + dist_pt_dep + dist_pt_arr +
                                   educ_res + educ_dep + educ_arr +
                                   intersec_res + intersec_dep + intersec_arr +
                                   dest_res + dest_dep + dest_arr +
                                   park_res + park_dep + park_arr +
                                   pdens_res + pdens_dep + pdens_arr +
                                   res_cour + dep_cour + arr_cour)

fit_tm <- randomForest(form.tm, data = egt, ntree = 1000)

# 2. Duration prediction model in EGT dataset
#      This later used in the simulation process to update
#      the duration of the trips after the mode is changed
#      Due to the large number of observations and
#      the continuous outcome variable, growing 1000 trees was not
#      possible due to calculation power. But the model was stable at
#      100 trees in terms of predictability. So , we decided to grow 150 trees

form_dur <- formula(duration_mn ~
                                    mode_trans1 +
                                    time_of_day + day_trip + rush_hour + dist_ld +
                                    age + homme + rvnu + emploi_sim + nivetude_sim +
                                    dist_train_dep + dist_metro_dep +
                                    dist_tram_dep + dist_bus_dep +
                                    dist_train_arr + dist_metro_arr +
                                    dist_tram_arr + dist_bus_arr +
                                    dist_train_res + dist_metro_res +
                                    dist_tram_res + dist_bus_res +
                                    dist_pt_res + dist_pt_dep + dist_pt_arr +
                                    educ_res + educ_dep + educ_arr +
                                    intersec_res + intersec_dep + intersec_arr +
                                    dest_res + dest_dep + dest_arr +
                                    park_res + park_dep + park_arr +
                                    pdens_res + pdens_dep + pdens_arr +
                                    res_cour + dep_cour + arr_cour )

fit_dur <- randomForest(form_dur, data = egt, ntree = 150)

# 3. T-MVPA prediction model in RECORD data set
#      This model is used for the data integration step and to update
#      the predicted T-MVPA during the simulation process after changing
#      the transportation mode and duration of a trip.

form_mv <- formula(mvpa_ep1m ~
                                   duration_mn + speed_ld +
                                   mode_trans1 +
```

```
                         time_of_day + day_trip + rush_hour + dist_ld +
                         age + homme + rvnu + emploi_sim + nivetude_sim +
                         dist_train_dep + dist_metro_dep +
                         dist_tram_dep + dist_bus_dep +
                         dist_train_arr + dist_metro_arr +
                         dist_tram_arr + dist_bus_arr +
                         dist_train_res + dist_metro_res +
                         dist_tram_res + dist_bus_res +
                         dist_pt_res + dist_pt_dep + dist_pt_arr +
                         educ_res + educ_dep + educ_arr +
                         intersec_res + intersec_dep + intersec_arr +
                         dest_res + dest_dep + dest_arr +
                         park_res + park_dep + park_arr +
                         pdens_res + pdens_dep + pdens_arr +
                         res_cour + dep_cour + arr_cour )

fit_mvp <- randomForest(form_mv, data = rec, ntree = 1000)


########################
# B. Prediction of MVPA for EGT trips (data integration step)
#    This will result in a predictive T-MVPA value for the observed trips
#    and is based on the above fitted random forest model.

# 1 Imputation of missing values in EGT dataset
#    The imputation will enable MVPA predictions for all trips
#    The impututing process are based on predictive mean matching models

# 1.1 Ordering the variables on the amount of missing values
#       while making sure id variables won't be used in the imputation

seq <- dimnames(md.pattern(egt[,5:ncol(egt), with = FALSE]))[[2]]
seq <- seq[-length(seq)]
seq <- c(c("trip_code", "resc", "depcom_res", "dciris_res"), seq)
egt <- egt[, seq, with = FALSE]
egt.ini <- copy(egt)
egt.ini[,':=' (trip_code = '1', resc = '1', depcom_res = 1, dciris_res = 1)]

# 1.2 Use mice() with the maximum number of iterations maxit set to zero.
#       This is a fast way to create the mids object called ini
#       containing the default settings.

ini <- mice(egt.ini, max = 0)
meth <- ini$meth
pred <- ini$pred
vis <- ini$vis

# 1.3 Actual imputation of the EGT dataset, only 1 dataframe retained

mi_data <- mice(egt, m = 1, maxit = 5, pred = pred, meth = meth, vis = vis)
```

```
egt.nomiss <- complete(mi_data)


# 2. Prediction of MVPA for each EGT trip
#      Using the new egt.nomiss dataset and
#      MVPA-prediction model on RECORD data
#      Note: the original not-imputed EGT dataset is used after this step
#      egt.nomiss is only used for these predictions

egt[, pred_mvpa := predict(fit_mvp, egt.nomiss)]


########################
# C Simulation step 1: Selection of trips
#      Here we give the example for the walking trips.
#      The procedure is similar for the other transportation mdoes
#      We created 100 selection variables (0,1) per scenario
#      by raising or lowering the probabilities,
#      in order to get the search mean probability of selection.
#      The selection variables will be written to separate files per scenario.

# 1. Probability estimates (= votes) from the random forest fitted
#      above added to the dataset

egt <- data.table(fit_tm$votes, egt)
setnames(egt, c('1. walking',   '2. bicycle', '3. motorized', '4. public'),
         c('walking', 'biking', 'motorized', 'public'))

# 2 The following function determines the trips to change from private motorised trips
# to public transport, walking or biking trips, dependent on the predefined number of
# trips for each mode.

mode.shift <- function(set1, nb.to.pub, nb.to.wal, nb.to.bik, n.sim){
  ###

  set <- copy(set1)
  set[, paste0('V', 1:n.sim) := as.character(NA)]
  set[, ':=' (walking_orig = walking, biking_orig = biking, public_orig = public)]

      # the number of private motorized trips that need to be changed into an
      # alternative mode
  nb.car.away <- nb.to.pub + nb.to.wal + nb.to.bik

  # begin for loop to create the different new mode_trans variables
  # 1 loop for each simulation (in the paper 100) within the scenario
      # the different simulations prevented results dependent on random selection
      # effects

  for(i in 1:n.sim){
```

```
set[mode_trans1 != '3. motorized', ':=' (walking = NA, biking = NA, public = NA)]
set[, new_mode_trans1 := '']

  # I introduced a while loop, since in rare cases trips can be selected to be
  # changed in multiple alternative modes. In that case, one alternative mode is
  # randomly selected, and a new loop provides a different trip for the
  # alternative mode that was not selected.

while(nrow(set[new_mode_trans1 != '']) < nb.car.away){

  # update the probability of being selected to
  nb.pub <- nb.to.pub - nrow(set[new_mode_trans1 == "4. public" ])
  nb.wal <- nb.to.wal - nrow(set[new_mode_trans1 == "1. walking"])
  nb.bik <- nb.to.bik - nrow(set[new_mode_trans1 == "2. bicycle"])

  set[, ':=' (sel.pub = 0, sel.wal = 0, sel.bik = 0)]

 # We calculate the shift of the probabilities, so that the mean probability equals
 # the proportion of trips we need to change

  if(nb.pub > 0){prop.pub <- nb.pub/nrow(set[new_mode_trans1 == '' &
mode_trans1 == "3. motorized"])}
  if(nb.wal > 0){prop.wal <- nb.wal/nrow(set[new_mode_trans1 == '' &
mode_trans1 == "3. motorized"])}
  if(nb.bik > 0){prop.bik <- nb.bik/nrow(set[new_mode_trans1 == '' &
mode_trans1 == "3. motorized"])}

  # the RF result variables that we want to shift (in the logit scale)
  if(nb.pub > 0){logit.var.pub <- logit(set[!is.na(public),   public],
adjust=.000025)}
  if(nb.wal > 0){logit.var.wal <- logit(set[!is.na(walking), walking],
adjust=.000025)}
  if(nb.bik > 0){logit.var.bik <- logit(set[!is.na(biking),   biking],   adjust=.000025)}

  # the ideal shift in proportion scale
  if(nb.pub > 0){shift.pub <- - optimize(f = fr, interval=c(-6, 4), y = prop.pub,
logit.var = logit.var.pub, tol = 0.000000001)$minimum}
  if(nb.wal > 0){shift.wal <- - optimize(f = fr, interval=c(-6, 4), y = prop.wal,
logit.var = logit.var.wal, tol = 0.000000001)$minimum}
  if(nb.bik > 0){shift.bik <- - optimize(f = fr, interval=c(-6, 4), y = prop.bik, logit.var
= logit.var.bik, tol = 0.000000001)$minimum}

  if(nb.pub > 0){set[!is.na(public),   sel.pub := sam5(public,   shift.pub)]}
  if(nb.wal > 0){set[!is.na(walking), sel.wal := sam5(walking, shift.wal)]}
  if(nb.bik > 0){set[!is.na(biking),   sel.bik := sam5(biking,   shift.bik)]}

  # if only one new transport mode is selected for a trip, than I assign that mode
  set[sel.pub == 1 & sel.wal == 0 & sel.bik == 0, new_mode_trans1 := '4. public']
  set[sel.pub == 0 & sel.wal == 1 & sel.bik == 0, new_mode_trans1 := '1.
walking']
```

```
        set[sel.pub == 0 & sel.wal == 0 & sel.bik == 1, new_mode_trans1 := '2.
bicycle']

        # if 2 or three transport modes are to a single, than one is selected randomly
        set[sel.pub == 1 & sel.wal == 1 & sel.bik == 0, new_mode_trans1 :=
sample(c('4. public', '1. walking'), .N, replace = TRUE)]
        set[sel.pub == 1 & sel.wal == 0 & sel.bik == 1, new_mode_trans1 :=
sample(c('4. public', '2. bicycle'), .N, replace = TRUE)]
        set[sel.pub == 0 & sel.wal == 1 & sel.bik == 1, new_mode_trans1 :=
sample(c('1. walking', '2. bicycle'), .N, replace = TRUE)]
        set[sel.pub == 1 & sel.wal == 1 & sel.bik == 1, new_mode_trans1 :=
sample(c('4. public', '1. walking', '2. bicycle'), .N, replace = TRUE)]

         # In case of multiple modes selected, a random selection is made for the trip
         # and the while loop starts again. This loop runs until we obtain exactly the
         # number of trips for each mode. Usually, 2 or 3 loops are enough.

        if(nrow(set[new_mode_trans1 == '4. public']) > nb.to.pub){
          selected <- nrow(set[sel.pub == 1 & new_mode_trans1 == '4. public'])
          too.much <- selected - nb.pub
          new.val <- c(rep('', too.much), rep('4. public', nb.pub))
          set[sel.pub == 1 & new_mode_trans1 == '4. public', new_mode_trans1 :=
sample(new.val, size = length(new.val), replace = FALSE)]
        }
        if(nrow(set[new_mode_trans1 == '1. walking']) > nb.to.wal){
          selected <- nrow(set[sel.wal == 1 & new_mode_trans1 == '1. walking'])
          too.much <- selected - nb.wal
          new.val <- c(rep('', too.much), rep('1. walking', nb.wal))
          set[sel.wal == 1 & new_mode_trans1 == '1. walking', new_mode_trans1 :=
sample(new.val, size = length(new.val), replace = FALSE)]
        }
        if(nrow(set[new_mode_trans1 == '2. bicycle']) > nb.to.bik){
          selected <- nrow(set[sel.bik == 1 & new_mode_trans1 == '2. bicycle'])
          too.much <- selected - nb.bik
          new.val <- c(rep('', too.much), rep('2. bicycle', nb.bik))
          set[sel.bik == 1 & new_mode_trans1 == '2. bicycle', new_mode_trans1 :=
sample(new.val, size = length(new.val), replace = FALSE)]
        }

        # clean up before the process is done again.
        set[, ':=' (sel.pub = NULL, sel.wal = NULL, sel.bik = NULL)]
        # get the probabilities deleted, so that the new loop doesn't take it into account
        set[mode_trans1 != '3. motorized' | new_mode_trans1 != '', ':=' (walking = NA,
biking = NA, public = NA)]
      }


      set[new_mode_trans1 == '', (paste0('V', i)) := mode_trans1]
      set[new_mode_trans1 != '', (paste0('V', i)) := new_mode_trans1]
```

```
      set[, ':=' (walking = walking_orig, biking = biking_orig, public = public_orig)]
      set[, new_mode_trans1 := '']
      print(i)
   }
   set
}

# The following for loop repeats the selection for three scenarios

for(i in 1:3){

   sel <- mode.shift(results, 1386*i,   688*i,   30*i, n.sim=100)

   path <- '…./your_directory/'
   filename <- paste0(path, "2. selection - AP ", i, ".RData")
   save(list = c('sel'), file = filename)

}


############################
# The following function 'select.pred' changes the values of the duration of the
# previously selected trips, and it then also updates the MVPA for these trips.


select.pred <- function(set, nsim){
   #########################
   set[, mode_trans_orig := mode_trans1]
   set[, duration_mn_orig := duration_mn]
   set[, speed_ld_orig := speed_ld]
   set[, pred_mvpa_orig := predict(fit.mvp, set)]

   ##############################
   var <- c(paste('V', 1:nsim, sep=""))
   pre.var <- c(paste('pre', 1:nsim, sep=""))

   # set up simulation function
   for(i in 1:nsim){
      set[, pre.var[i]] <- set[, pred_mvpa_orig]
      set[, 'mod.var'] <- set[, as.factor(var[i]), with = F]
      set[var[i] != mode_trans_orig,   'mode_trans1' := mod.var]
      set[var[i] != mode_trans_orig, duration_mn := NA]
      set[var[i] != mode_trans_orig, speed_ld := NA]

          # in the following three lines, the duration of the trips is predicted, then the
        # speed and finally the MVPA, each time based on a random forest model
      set[var[i] != mode_trans_orig, duration_mn := as.integer(predict(fit.dur, set[var[i]
!= mode_trans_orig]))]
      set[var[i] != mode_trans_orig, speed_ld := dist_ld/(duration_mn/60)]
      set[var[i] != mode_trans_orig, pre.var[i] := predict(fit.mvp, set[var[i] !=
```

```
mode_trans_orig])]

      set[var[i] != mode_trans_orig, duration_mn := duration_mn_orig]
      set[var[i] != mode_trans_orig, speed_ld := speed_ld_orig]
      set[var[i] != mode_trans_orig, mode_trans1 := mode_trans_orig]
      if((i %% 10) == 0) print(i)
   }

   set
}

# This for loop applies the select.pred function to the three scenarios

for(sim in 1:3){
   pathin = ‘…./your_directory/’
   load(file = paste0(pathin, "2. selection - AP ", sim, ".RData"))

   sel <- select.pred(sel, 100)

   pathout <- ‘…./your_directory/’
   save(list = c('sel'), file = paste0(pathout, "1 simulations - AP ", sim, ".RData"))
}




#######################
# E Aggregate at day level
#    T-mvpa was measured at trip-level, but reported at day-level, i.e. we
#    reported the accumulated T-mvpa during the day.

# 1. The aggr_day function aggregates the data set to the day-level
#      by calculating the mean T-MVPA per day and per transportation mode per day
#      and this for each of the 100 simulation within a scenario

aggr.day <- function(set_orig, nsim){
   set <- copy(set_orig)
   a <- unlist(strsplit(as.character(set$trip_code), '_'))
   men <- a[seq(1,length(a), 3)]
   per <- a[seq(2,length(a), 3)]
   set$person <- paste(men, per, sep='_')

   # total day variables
   setkey(set, person)
   set[,nb.ind := 1]

   var1 <- c(pre.var, 'nb.ind')
   var2 <- c(day.var, 'nb_trips')
   set[,     (var2) := lapply(.SD, sum, na.rm=TRUE), by=person, .SDcols=var1]
```

```
  # mvpa per type of mode_trans per person per simulation
  mod.var <- c(paste0('V', 1:nsim))

  for(i in 1:nsim){
    set[, 'mode_sim'] <- set[,mod.var[i], with=FALSE]
    set[, 'pred_sim'] <- set[,pre.var[i], with=FALSE]
    setkey(set, person, mode_sim)

    # summary datasets
    mvpa_by_mt <- set[, sum(pred_sim), by=list(person,mode_sim)]
    setkey(mvpa_by_mt, person, mode_sim)
    out <- mvpa_by_mt[CJ(unique(person), unique(mode_sim))][, as.list(V1),
by=person]

    setnames(out, paste('V', 1:4, sep=''),
             paste("MVPA_", c("walking", "biking", "PM", "PT"), "_sim", i, sep=''))

    set <- merge(set, out, by='person')

    # number of trips

    setkey(set, person, mode_sim)
    nb_by_mt <- set[, sum(nb.ind), by=list(person,mode_sim)]

    setkey(nb_by_mt, person, mode_sim)
    out <- nb_by_mt[CJ(unique(person), unique(mode_sim))][, as.list(V1),
by=person]

    setnames(out, paste('V', 1:4, sep=''),
             paste("nb_", c("walking", "biking", "PM", "PT"), "_sim", i, sep=''))

    set <- merge(set, out, by='person')

    # accumulated distance of trips changed

    setkey(set, person, mode_sim)
    dist_by_mt <- set[, sum(dist_ld), by=list(person,mode_sim)]

    setkey(dist_by_mt, person, mode_sim)
    out <- dist_by_mt[CJ(unique(person), unique(mode_sim))][, as.list(V1),
by=person]

    setnames(out, paste('V', 1:4, sep=''),
             paste("dist_", c("walking", "biking", "PM", "PT"), "_sim", i, sep=''))

    set <- merge(set, out, by='person')

    if((i%%10)==0) print(i)

  }
```

```
  ###################################
  # aggregate to day level
  set <- unique(setkey(set, person), by='person')

  # delete variables in egtnt, so it has no variables not in egt
  varnt <- names(egtnt)[which(names(egtnt) %in% names(set))]
  egtnt2 <- egtnt[,varnt, with=FALSE]

  # Merge set and setnt
  set <- rbindlist(list(set, egtnt2), use.names=TRUE, fill=TRUE)


  # T-MVPA variables with NA values are actually = 0
  replacena <- function(var){var <- replace(var, is.na(var), 0)}
  a <- rep(1:nsim,4) ; b <- a[order(a)]

  var <- c(day.var, 'nb_trips',
           paste0(rep(paste0("MVPA_", c("walking", "biking", "PM", "PT"), "_sim"),
nsim), b),
           paste0(rep(paste0("nb_",    c("walking", "biking", "PM", "PT"), "_sim"),
nsim), b),
           paste0(rep(paste0("dist_", c("walking", "biking", "PM", "PT"), "_sim"),
nsim), b))
  set[, (var) := lapply(.SD, replacena),.SDcols=var]

  # delete some var
  var <- c(paste0('V',     1:nsim),
           paste0('pre',   1:nsim),
           c("mode_trans1", "dest_arr", "pdens_arr", "nivetude_sim", "intersec_arr",
"arr_cour",
             "dist_metro_arr", "dist_train_arr", "dist_tram_arr", "dist_bus_arr",
             "dist_pt_arr", "dest_dep", "pdens_dep", "intersec_dep", "dep_cour",
"dist_metro_dep",
             "dist_train_dep", "dist_tram_dep", "dist_bus_dep", "dist_pt_dep",
             "dist_ld", "duration_mn", "speed_ld", "park_arr", "park_res", "park_dep",
             "educ_arr", "educ_dep", "educ_res",   "mode_sim", "pred_sim"))

  set[, (var) := NULL]
  set
}

# This for loop applies the 'aggr.day' function to the three scenarios
for(sim in 1:3){
  # selection file for transportation mode
  pathin <- '…./your_directory/'
  load(paste0(pathin, "1 simulations - AP ", sim, ".RData"))

  sel <- aggr.day(sel, nsim)
```

```
   pathout <- '…./your_directory/'
   save(list = c('sel'),
          file = paste0(pathout, "1 simulations - AP ", sim, ".RData"))
}


# 2. This code reads in a dataset with people include in the EGT dataset
#      that did not perform any trip. Therefore, they were not included
#      in the EGT dataset at trip-level. But they need to be in the
#      aggregated dataset

p.or <- '…./your_directory/'
egtnt <- data.table(read.csv(paste(p.or, '2. egt no trips.csv', sep="")))

# egt T-MVPA variables to 0 for people with no trips
var <- c('mvpa.day', 'nb_trips',
             paste0("MVPA_", c("walking", "biking", "PM", "PT")),
             paste0("MIN_",   c("walking", "biking", "PM", "PT")),
             paste0("nb_",     c("walking", "biking", "PM", "PT")))
egtnt[, (var) := 0]
egtnt[, egtnt := 1]

path <- '…./your_directory/'
save(list = c('egtnt'),
       file = paste0(path, "no trip people.RData"))




########################
# F Multiple imputation of EGT day-level dataset
#    Some variables had missing values in the EGT-dataset
#    Therefore, we created a multiple imputation dataset with 5 datasets.
#    This dataset is created for the original EGT dataset, and then linked
#    to the simulated datasets. So, all simulated datasets and the original
#    dataset the imputations of the independent variables are equal.
#    Note: In this particular study, only the imputations for educational level
#    are important since the other variables are not used in the reported results.

# 1. Ordering the variables on the amount of missing values
#    while making sure id variables won't be used in the imputation

seq <- dimnames(md.pattern(egt[,6:ncol(egt), with = FALSE]))[[2]]
seq <- seq[-length(seq)]
seq <- c(c('person', 'resc', 'depcom_res', 'dciris_res', 'over'), seq)
egt <- egt[, seq, with = FALSE]

# 2. Use mice() with the maximum number of iterations maxit set to zero.
#    This is a fast way to create the mids object called ini
#    containing the default settings.
```

```
egt.ini <- copy(egt)

egt.ini[,':=' (person = '1', resc = '1',   depcom_res = 1,
                     dciris_res = 1, over = 1)]
ini <- mice(egt.ini, max = 0, meth = 'rf')

meth <- ini$meth
meth[c("person", "resc", "depcom_res", "dciris_res",
         "mvpa_day_int", "age_10", "homme",   "res_cour",   "pos_motorized",
         "weekday",   "min_day",   "MIN_walking",
         "MIN_biking", "MIN_PM", "MIN_PT", "nb_trips",
         "nb_walking", "nb_biking",   "nb_PM",   "nb_PT", "over",
         'intersec_res_1000',   'pdens_res')] <- ""
pred <- ini$pred
vis <- ini$vis

# 3. Actual imputation of EGT dataset
#    Method is Random Forest, 5 imputations, 100 trees per imputation

mi_rf <- mice(egt, m = 5, pred = pred, meth = meth, ntree = 100, vis = vis)

#######################
# G Linking the simulation datasets to the multiple imputation dataset

# 1. This step defines the names of the variables to be calculated and
#      added to the multiple imputation dataset

nsim <- 100
var_sim <- paste0('mvpa_day',1:nsim)

var_mvpa_wal <- paste0('MVPA_walking_sim',1:nsim)
var_mvpa_bik <- paste0('MVPA_biking_sim',1:nsim)
var_mvpa_car <- paste0('MVPA_PM_sim',1:nsim)
var_mvpa_pub <- paste0('MVPA_PT_sim',1:nsim)

var_nb_wal <- paste0('nb_walking_sim',1:nsim)
var_nb_bik <- paste0('nb_biking_sim',1:nsim)
var_nb_car <- paste0('nb_PM_sim',1:nsim)
var_nb_pub <- paste0('nb_PT_sim',1:nsim)

var_dis_wal <- paste0('dist_walking_sim',1:nsim)
var_dis_bik <- paste0('dist_biking_sim',1:nsim)
var_dis_car <- paste0('dist_PM_sim',1:nsim)
var_dis_pub <- paste0('dist_PT_sim',1:nsim)

# This step add
# 2. This for loop reads in all datasets at day-level
#      (Note: day-level = person-level, since there is one day per perons)
#      Then, the mean MVPA total and per day are calculated for the
```

```
#      simulation dataset and added to the multiple imputation dataset.


for(sim in 1:3){
   # selection file for transportation mode
   pathin <- '…./your_directory/'
   load(paste0(pathin, "1 simulations - AP ", sim, ".RData"))

   sel <- sel[order(as.character(sel$person)),]
   mi.rf$data[,paste0('mvpa_day_ap_', sim)] <- sel[, rowMeans(.SD), .SDcols=
var_sim]

   mi.rf$data[,paste0('mvpa_wal_day_ap_', sim)] <- sel[, rowMeans(.SD), .SDcols=
var_mvpa_wal]
   mi.rf$data[,paste0('mvpa_bik_day_ap_', sim)] <- sel[, rowMeans(.SD), .SDcols=
var_mvpa_bik]
   mi.rf$data[,paste0('mvpa_car_day_ap_', sim)] <- sel[, rowMeans(.SD), .SDcols=
var_mvpa_car]
   mi.rf$data[,paste0('mvpa_pub_day_ap_', sim)] <- sel[, rowMeans(.SD), .SDcols=
var_mvpa_pub]

   mi.rf$data[,paste0('nb_wal_day_ap_', sim)] <- sel[, rowMeans(.SD), .SDcols=
var_nb_wal]
   mi.rf$data[,paste0('nb_bik_day_ap_', sim)] <- sel[, rowMeans(.SD), .SDcols=
var_nb_bik]
   mi.rf$data[,paste0('nb_car_day_ap_', sim)] <- sel[, rowMeans(.SD), .SDcols=
var_nb_car]
   mi.rf$data[,paste0('nb_pub_day_ap_', sim)] <- sel[, rowMeans(.SD), .SDcols=
var_nb_pub]

   mi.rf$data[,paste0('dist_wal_day_ap_', sim)] <- sel[, rowMeans(.SD), .SDcols=
var_dis_wal]
   mi.rf$data[,paste0('dist_bik_day_ap_', sim)] <- sel[, rowMeans(.SD), .SDcols=
var_dis_bik]
   mi.rf$data[,paste0('dist_car_day_ap_', sim)] <- sel[, rowMeans(.SD), .SDcols=
var_dis_car]
   mi.rf$data[,paste0('dist_pub_day_ap_', sim)] <- sel[, rowMeans(.SD), .SDcols=
var_dis_pub]
}
##################################################
# H. Results
#      To calculate the results, the means had to be calculated per
#      multiple imputed dataset and then pooled. Function mean_pool
#      calculated the overall means, mean_pool_g calculated
#      the means per category of a variable, here educational level.

mean.pool <- function(mi.data, var, id, gvar=NULL, group=NULL, change=NULL){
   m <- mi.data$m
   Q <- U <- rep(NA, m)
   for (i in 1:m) {
```

```
      set <- complete(mi.data, i)
      if(!is.null(change)){
        set <- set[which(set[,change] == 1),]
      }
      if(is.null(group)){
        var1 <- set[,var]
      } else {
        var1 <- set[which(set[,gvar]==group),var]
      }
      Q[i] <- mean(var1)
      U[i] <- var(var1) / nrow(complete(mi.data, i))   # (standard error of estimate)^2
    }
    me <- round(pool.scalar(Q, U, n = nrow(set), k = 1)$qbar,2)
    se <- round(pool.scalar(Q, U, n = nrow(set), k = 1)$ubar,2)
    data.table(sim=id, TMVPA=me)
}


mean.pool.g <- function(mi.data, var, gvar, id, change=NULL){
    m <- mi.data$m
    set <- complete(mi.data)
    lev <- levels(set[,gvar])
    lev2 <- gsub(" ", "_", lev)
    lev2 <- gsub("-", "", lev2)
    lev2 <- gsub("__", "_", lev2)
    lev2 <- gsub("[.]", "", lev2)
    lev2 <- paste0('Educ', 1:3)
    res <- data.table(level = lev, mean = rep(as.numeric(NA), length(lev)))
    for(i in 1:length(lev)){
      pres <- mean.pool(mi.data, var, id, gvar, lev[i], change)
      res[level == lev[i], mean := pres[1,TMVPA]]
    }
    res2 <- data.table(t(res[,mean]))
    setnames(res2, paste0('V', 1:3), lev2)
    #res2[, sim := id]
    res2
}

# examples
mean.pool(mi_rf, 'mvpa_day', 'original')
mean.pool.g(mi_rf, 'mvpa_day', 'nivetude_sim', 'original')



#############
# References to R-packages used
#    A. Liaw and M. Wiener (2002). Classification and Regression by
#    randomForest. R News 2(3), 18--22.
#
#    M Dowle, A Srinivasan, T Short, S Lianoglou with contributions
```

```
#	from R Saporta and E Antonyan (2015). data.table: Extension of
#	Data.frame. R package version 1.9.6.
#	https://CRAN.R-project.org/package=data.table

#	Stef van Buuren, Karin Groothuis-Oudshoorn (2011). mice: Multivariate
#	Imputation by Chained Equations in R. Journal of Statistical Software,
#	45(3), 1-67. http://www.jstatsoft.org/v45/i03/

#	Matt Dowle and Arun Srinivasan (2017). data.table: Extension of `data.frame`. R
#	package version 1.10.4. https://CRAN.R-project.org/package=data.table
```
